# Variation in the Form of Pavlovian Conditioned Approach Behavior among Outbred Male Sprague-Dawley Rats from Different Vendors and Colonies: Sign-Tracking vs. Goal-Tracking

**DOI:** 10.1371/journal.pone.0075042

**Published:** 2013-10-01

**Authors:** Christopher J. Fitzpatrick, Shyam Gopalakrishnan, Elizabeth S. Cogan, Lindsay M. Yager, Paul J. Meyer, Vedran Lovic, Benjamin T. Saunders, Clarissa C. Parker, Natalia M. Gonzales, Emmanuel Aryee, Shelly B. Flagel, Abraham A. Palmer, Terry E. Robinson, Jonathan D. Morrow

**Affiliations:** 1 Neuroscience Graduate Program, University of Michigan, Ann Arbor, Michigan, United States of America; 2 Department of Human Genetics, University of Chicago, Chicago, Illinois, United States of America; 3 Biopsychology Graduate Program, University of Michigan, Ann Arbor, Michigan, United States of America; 4 Department of Psychology, University of Michigan, Ann Arbor, Michigan, United States of America; 5 Department of Psychiatry, University of Michigan, Ann Arbor, Michigan, United States of America; 6 Department of Psychiatry and Behavioral Neuroscience, University of Chicago, Chicago, Illinois, United States of America; Sapienza University of Rome, Italy

## Abstract

Even when trained under exactly the same conditions outbred male Sprague-Dawley (SD) rats vary in the form of the Pavlovian conditioned approach response (CR) they acquire. The form of the CR (i.e. sign-tracking vs. goal-tracking) predicts to what degree individuals attribute incentive salience to cues associated with food or drugs. However, we have noticed variation in the incidence of these two phenotypes in rats obtained from different vendors. In this study, we quantified sign- and goal-tracking behavior in a reasonably large sample of SD rats obtained from two vendors (Harlan or Charles River), as well as from individual colonies operated by both vendors. Our sample of rats acquired from Harlan had, on average, more sign-trackers than goal-trackers, and vice versa for our sample of rats acquired from Charles River. Furthermore, there were significant differences among colonies of the same vendor. Although it is impossible to rule out environmental variables, SD rats at different vendors and barriers may have reduced phenotypic heterogeneity as a result of genetic variables, such as random genetic drift or population bottlenecks. Consistent with this hypothesis, we identified marked population structure among colonies from Harlan. Therefore, despite sharing the same name, investigators should be aware that important genetic and phenotypic differences exist among SD rats from different vendors or even from different colonies of the same vendor. If used judiciously this can be an asset to experimental design, but it can also be a pitfall for those unaware of the issue.

## Introduction

Even the most experimentally reproducible behaviors show consistent inter-individual variation. Understanding the sources of this variation allows better control of experimental conditions in order to reduce “noise” and increase the probability of obtaining replicable results. Environmental factors, such as lighting, odors, and housing conditions, are routinely recognized as important variables and standardized in behavioral experiments. Genetic factors are also well-known, and in animals studies are typically controlled by limiting analyses to a particular strain or stock of animals.

Both inbred and outbred rats can be used in studies investigating rodent behavior, and each has a different level of genetic diversity. Inbred rats, such as Lewis and Fischer, are referred to as “strains” and are the product of deliberate inbreeding among siblings for a minimum of twenty generations [1]. Individuals from a given inbred strain are considered isogenic, or genetically identical. Each inbred strain has a unique genotype that has become fixed over successive generations of inbreeding, and the behavioral phenotype is correspondingly constrained. In contrast, outbred rats, referred to as “stocks”, are maintained using breeding schemes intended to preserve genetic diversity. Outbred stocks, such as Sprague-Dawley (SD), are often popular research models for translational studies of behavior and pharmacology due to their genetic diversity, which may more closely mirror the genetic and behavioral heterogeneity observed in human populations [2].

With increased genetic diversity comes an increased potential for stable behavioral differences between reproductively isolated populations, such as rats from different vendors or even different colonies within the same vendor. Vendor differences in behavioral, pharmacological, and physiological measures have been repeatedly identified in the SD outbred stock [3], [4], [5], [6], [7], [8], [9], [10] as well as in Wistar-Kyoto [11] and Wistar [12], [13], [14] stocks. In addition, colony differences in the learned helplessness behavioral measure have been demonstrated in SD rats acquired from different colonies within Harlan [15]. Therefore, vendor and colony differences are commonplace, and may produce reliable variation in a variety of experimental measures.

Our laboratory focuses on individual differences in Pavlovian conditioned approach (PCA) as a model of the attribution of incentive salience to predictive cues. PCA is a learned behavior that develops when a conditioned stimulus (CS; a lever) precedes a response-independent unconditioned stimulus (US; a food pellet delivered into a pellet magazine). If the CS and US are physically separated, different phenotypes can manifest: sign-tracking (CS-directed behavior), goal-tracking (US-directed behavior), and an intermediate phenotype expressing both behaviors. Development of a sign-tracking response involves attribution of incentive salience to the CS, transforming the CS from a mere predictor of the US into an attractive, rewarding stimulus in its own right [16], [17]. Individuals that sign-track to discrete, localizable conditioned food cues are also more likely to work for drug-associated cues [18], and are more likely to reinstate responding for food or drug cues after extinction [19], [20]. Thus, sign-tracking appears to indicate a general tendency to attribute incentive salience to discrete conditioned cues, which may render the individual more susceptible to maladaptive cue-driven behaviors, such as drug addiction and binge eating. Because behavioral measures such as PCA potentially vary between populations, we analyzed the PCA behavior in a relatively large sample (n = 557) of SD rats pooled from several studies in order to determine how PCA phenotypes differ based on vendor and colony. We also tested for population structure among three colonies at Harlan to determine whether the colonies are genetically distinct from one another.

## Materials and Methods

### Animals

Adult male Sprague-Dawley rats (250–300 g) were purchased from either Charles River (n = 115) or Harlan (n = 442). Furthermore, these animals were specifically acquired from different colonies. SD rats from Charles River were selected from colonies P03 (n = 30; Portage, MI) and P09 (n = 85; Portage, MI); SD rats from Harlan were selected from colonies 206 (n = 199; Haslett, MI), 208A (n = 150; Frederick, MD), and 217 (n = 93; Indianapolis, IN). Tail samples from a subset of the Harlan animals were subjected to genetic analysis. Animals were maintained on a 12∶12-hr light/dark cycle, and food and water were available *ad libitum* for the duration of experimentation. All procedures were approved by the University Committee on the Use and Care of Animals (University of Michigan; Ann Arbor, MI).

### Pavlovian Conditioned Approach: Apparatus

Operant conditioning modular chambers (24.1 cm width×20.5 cm depth×29.2 cm height; MED Associates, Inc.; St. Albans, VT) were used for Pavlovian conditioning. Each chamber was located in a sound-attenuating cubicle equipped with a ventilation fan to provide ambient white noise. Each chamber was equipped with a pellet magazine, an illuminated, retractable lever (counterbalanced on the left or right of the pellet magazine), and a red house light on the wall opposite of the pellet magazine. When inserted into the chamber, the retractable lever was illuminated by a LED light within the lever housing. A pellet dispenser delivered banana-flavored food pellets into the pellet magazine. An infrared sensor inside the pellet magazine measured head entries to the pellet magazine.

### Pavlovian Conditioned Approach: Procedure

For two days prior to pretraining, rats were familiarized with banana-flavored food pellets (45 mg; Bioserv; Frenchtown, NJ) in their home cages. Rats were then placed into the operant chambers for one or two pretraining sessions. Initial studies used two pretraining sessions on successive days, but the second session was eliminated in subsequent experiments because analysis indicated no difference in behavior between animals who received one vs two pretraining sessions (Fig. S1; t = 1.771, p = .077). During pretraining sessions, the red house-light remained on but the lever was retracted. Fifty food pellets were delivered on a variable interval (VI) 30-s schedule (i.e., one food pellet was delivered on average every 30 s, but actual delivery varied between 0–60 s). All rats consumed all the food pellets by the end of the first or second pretraining session, after which rats started PCA training. Each trial during a test session consisted of extension of the illuminated lever (CS) into the chamber for 8 s on a VI 90-s schedule. Retraction of the lever was immediately followed by the response-independent delivery of one food pellet (US) in the pellet magazine. Each test session consisted of 25 trials of CS-US pairings, resulting in a total session length of approximately 40 m. Each rat consumed all the food pellets that were delivered. The rats were tested by different experimenters as part of ongoing studies from February to June 2012. Although subsets of animals were used in different studies, the initial Pavlovian training procedure was the same in all cases, and these are the data reported here. In no case did any experimental manipulation precede Pavlovian training.

### Genotyping-by-sequencing and Population Structure

We used genotyping-by-sequencing (GBS), which is a reduced representation sequencing approach, to obtain genotypes at single nucleotide polymorphisms (SNPs) that were approximately evenly spaced throughout the rat genome. GBS was performed on 66 SD rats from 3 Harlan colonies: 28 from Haslett (206), 4 from Frederick (208A) and 34 from Indianapolis (217) [21], [22]. GBS libraries were prepared by digesting genomic DNA with *PstI* and annealing indexed adapters to the resulting overhangs, thus all regions that were proximal to *PstI* restriction sites were sequenced; a subset of those regions contained SNPs. We performed discovery of SNPs and called genotypes using *bwa* [23] and *samtools* [24]. This procedure yielded 2,256 high confidence SNPs that had minor allele frequencies greater than 0.2.

Using these genotype data we conducted a test for population stratification using the program *structure* [25] to determine whether genetic differences between the colonies existed; such differences may explain the phenotypic differences that we observed between these three Harlan colonies.

### Statistical Analysis

PCA behavior was scored using an index that averages the frequency, probability, and latency of lever presses (sign-tracking response) and magazine entries (goal-tracking response) during CS presentations within a session [26]. Briefly, we averaged together the response bias (i.e., number of lever presses and magazine entries for a session; [lever presses – magazine entries]/[lever presses+magazine entries]), probability difference (i.e., proportion of lever presses or magazine entries; [lever press probability – magazine entry probability]/100), and latency score (i.e., average latency to perform a lever press or magazine entry during a session; [lever press latency – magazine entry latency]/8). The index scores behavior from −1.0 (absolute goal-tracking) to +1.0 (absolute sign-tracking), with 0 representing no bias. All statistical analyses were performed using SPSS (Version 16; IBM, Inc.), which automatically adjusts for unequal sample sizes. Differences between two groups were analyzed using Student’s t-tests. Repeated measures were analyzed using a linear mixed model with an autoregressive (AR1) covariance structure.

## Results

**Figure 1:**
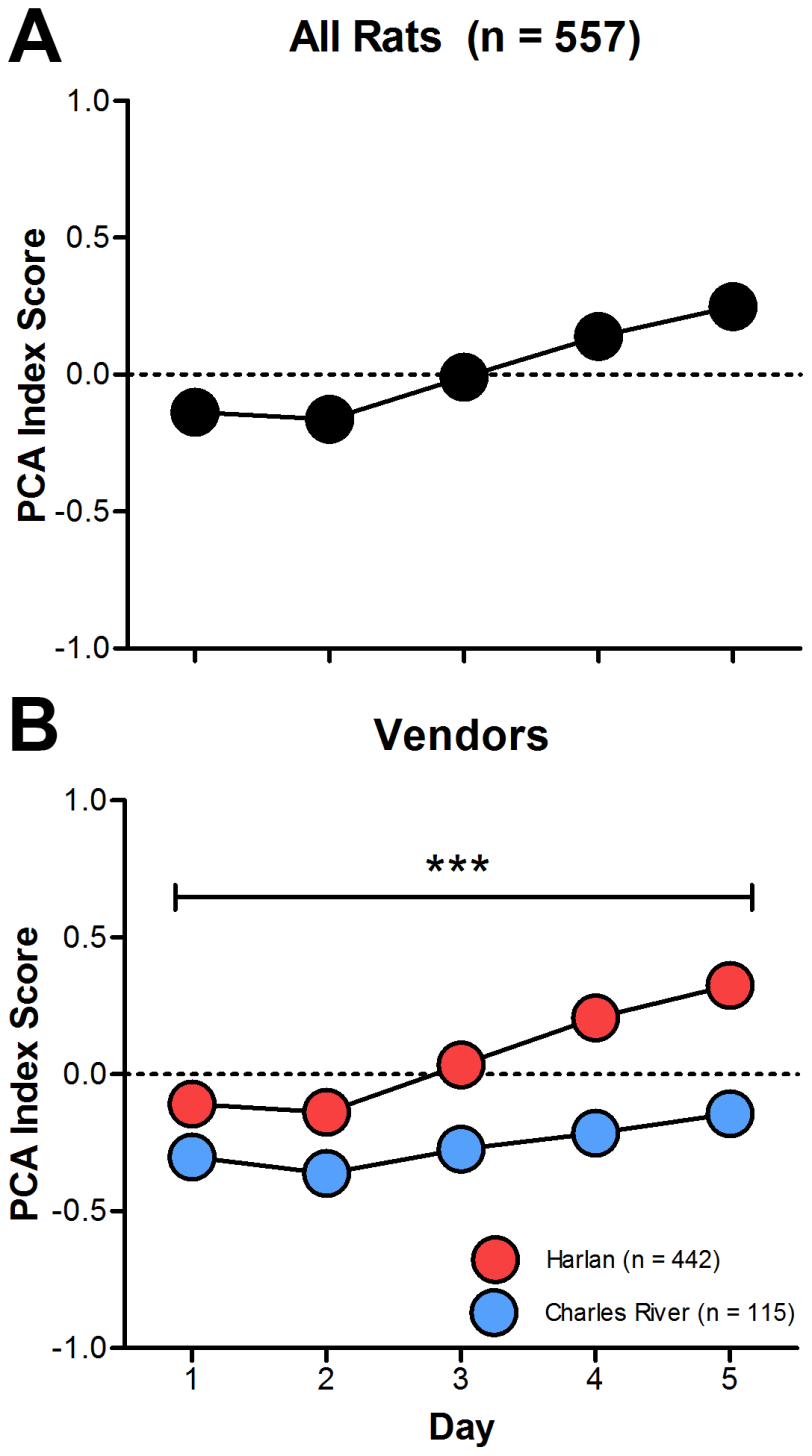
Pavlovian conditioned approach index scores between vendors. PCA scores (mean+S.E.M.) across five daily sessions of training for (A) all rats (n = 557) as well as (B) rats subdivided by vendor (Harlan, n = 442; Charles River, n = 115). Note that error bars are included in the figure, but they are so small that the data symbols obscure them. *** - p<0.001.

**Figure 2:**
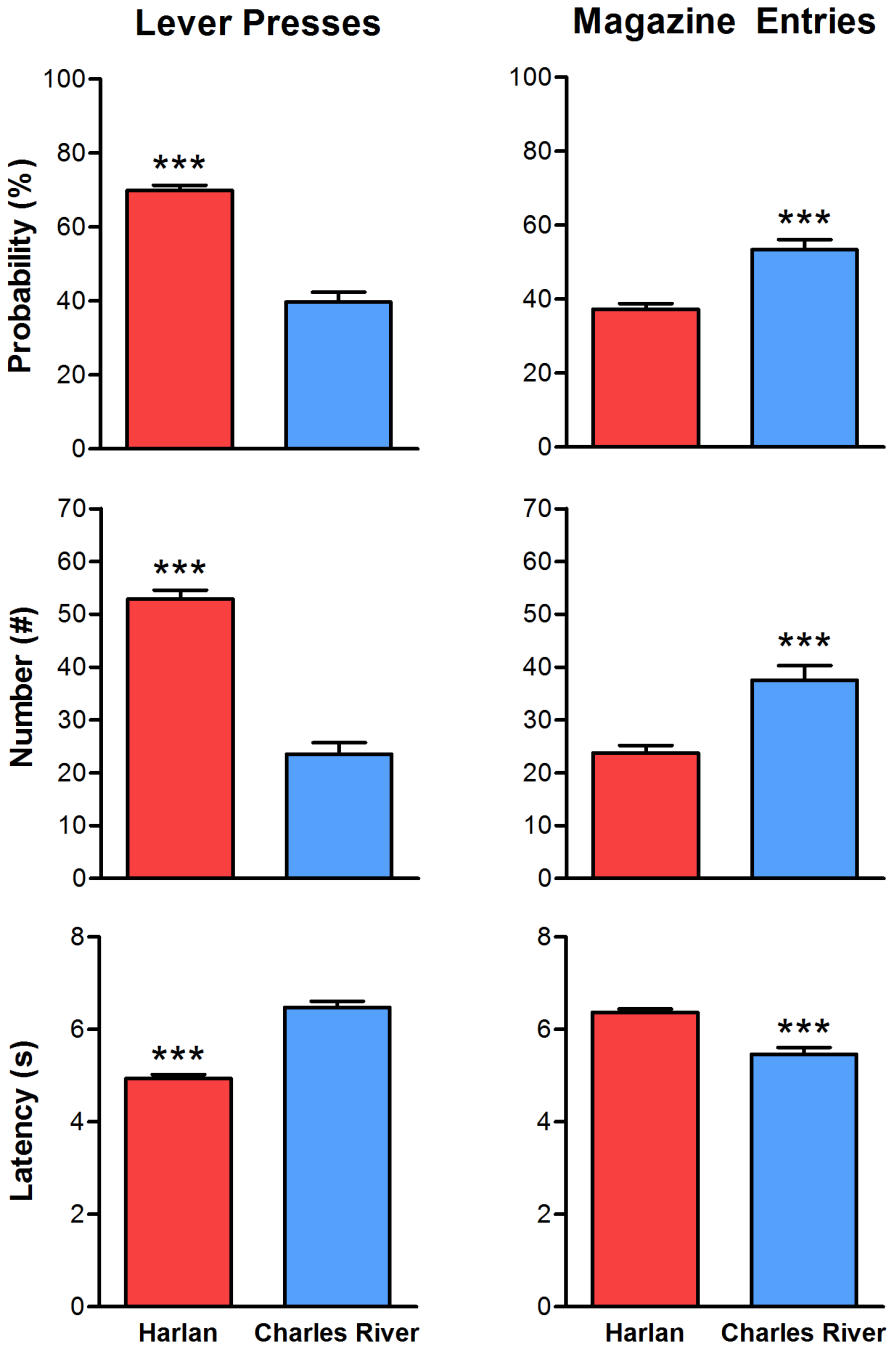
Pavlovian conditioned approach variables between vendors. PCA variables (mean+S.E.M.) averaged across the last two sessions. Variables included the number, probability, and latency of lever presses (sign-tracking conditioned response) and magazine entries (goal-tracking conditioned response) during US presentation. *** - p<0.001.

**Figure 3:**
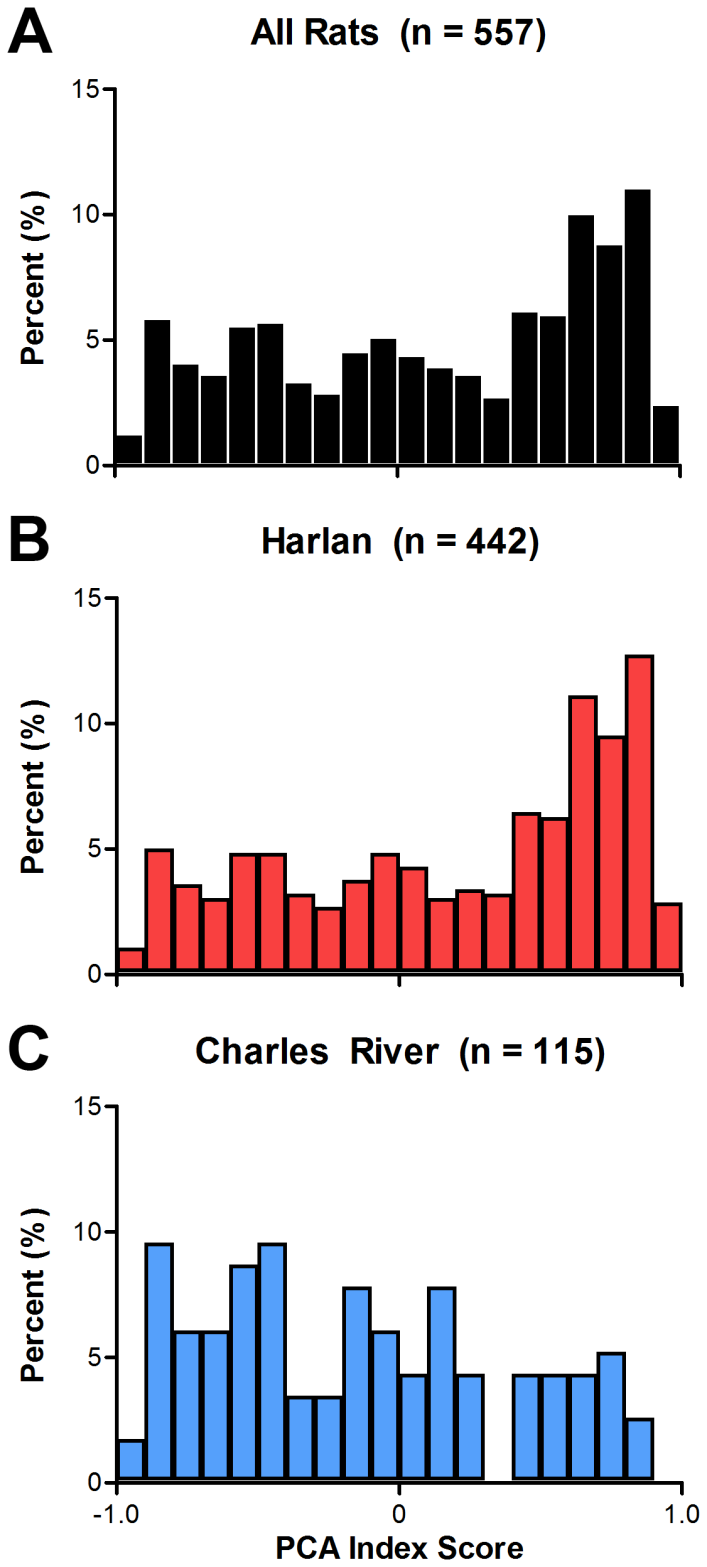
Distribution of Pavlovian conditioned approach index scores between vendors. The percent distribution of PCA scores, averaged from the last two sessions of training, of (A) all rats as well as the total sample subdivided into rats acquired from (B) Harlan (n = 442) or (C) Charles River (n = 115). Rats are binned by PCA score (−1.0 to +1.0) in increments of 0.10.

Figure 1 shows a comparison of PCA behavior across five days of training in rats obtained from two different vendors, Harlan and Charles River. It can be seen that rats supplied from Harlan had significantly higher PCA index scores (i.e., more sign-tracking behavior) than rats supplied from Charles River over the five training sessions (Fig. 1; main effect of vendor; F_(1,642.21)_ = 55.63, p = 2.85×10^−13^). Furthermore, by the last two days of training rats from Harlan had positive PCA scores whereas rats from Charles River had, on average, negative PCA scores (i.e., more goal-tracking). Figure 2 shows the averages (over the last two sessions) of the individual variables used to calculate PCA index scores: number, probability, and latency of lever presses and magazine entries. Harlan rats performed more lever presses during the CS period (t = 60.87, p = 3.05×10^−14^) with a shorter latency (t = 61.58, p = 2.20×10^−14^) and higher probability (t = 79.03, p = 8.55×10^−18^). In contrast, rats from Charles River performed more magazine entries during the CS period (t = 18.22, p = 2.32×10^−5^) with a shorter latency (t = 24.82, p = 8.41×10^−7^) and higher probability (t = 20.39, p = 7.70×10^−6^). When PCA index scores are plotted as a histogram, it is readily apparent that more sign-tracking rats came from Harlan whereas more goal-tracking rats came from Charles River (Fig. 3; t-test of PCA index scores; t = 140.76, p = 4.29×10^−29^).

**Figure 4:**
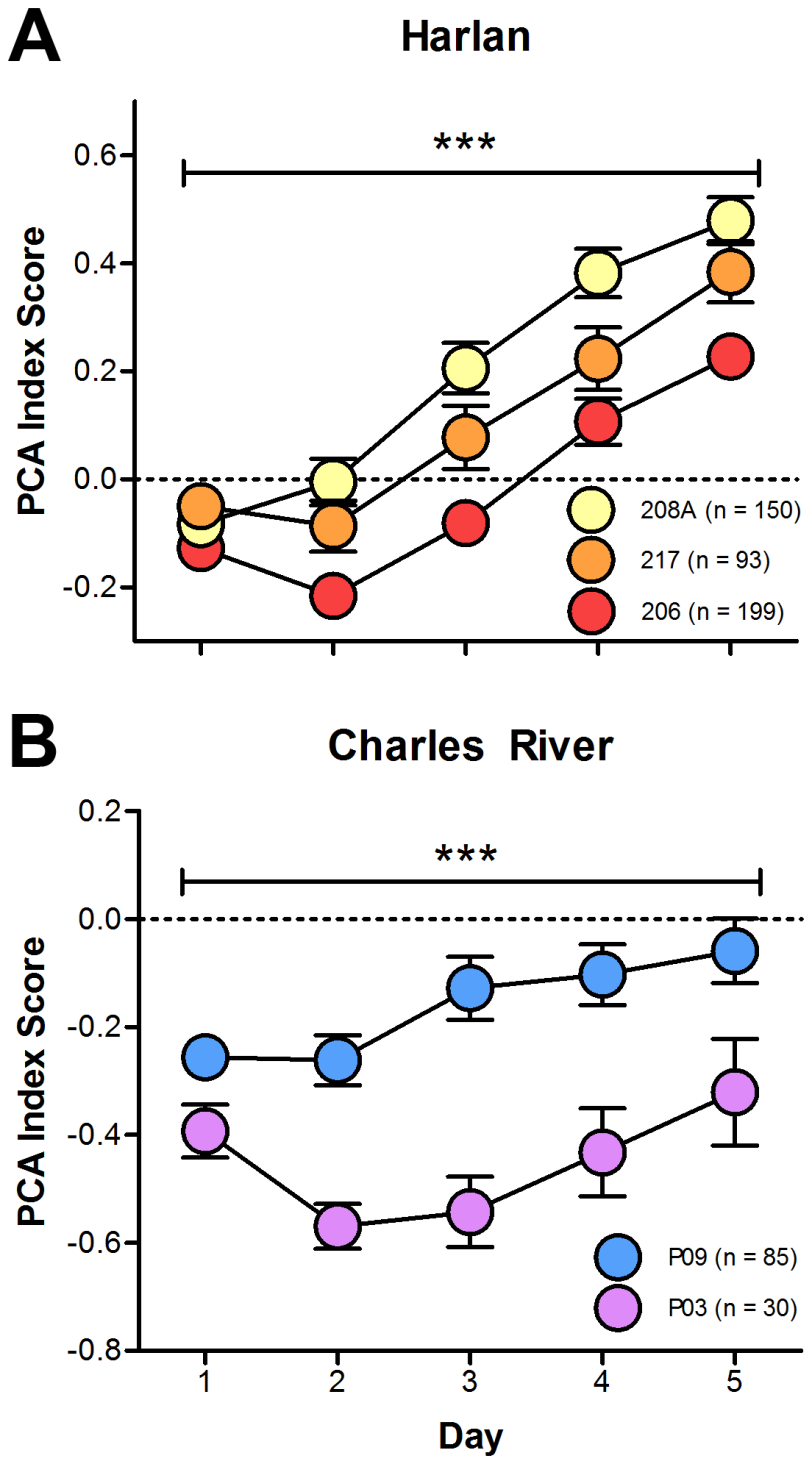
Pavlovian conditioned approach scores between colonies. PCA scores (mean+S.E.M.) across five daily sessions of training subdivided by different barriers within (A) Harlan and (B) Charles River. *** - p<0.001.

In addition to differences in PCA behavior of rats supplied from different vendors, differences were also observed in rats supplied from individual barriers of each vendor. Harlan rats from barriers 206 (Haslett, MI), 208A (Frederick, MD), and 217 (Indianapolis, IN) displayed significant differences in their respective PCA index scores (Fig. 4*A*; main effect of barrier; F_(2,506.46)_ = 7.86, p = 4.34×10^−4^). Rats from these barriers differed in the number, latency, and probability of lever presses (data not shown; number: F_(2,474.82)_ = 9.57, p = 8.43×10^−5^; latency: F_(2,486.19)_ = 4.875, p = 0.008; probability: F_(2,481.51)_ = 3.922, p = 0.02). Correspondingly, rats from these barriers also had different numbers, latencies, and probabilities of magazine entries during the CS (data not shown; number: F_(2,567.84)_ = 5.40, p = 0.005; latency: F_(2,577.98)_ = 5.82, p = 0.003; probability: F_(2,550.16)_ = 5.51, p = 0.004).

Charles River rats from barrier P03 (Portage, MI) had lower PCA index scores (i.e., more goal-tracking behavior) than rats from barrier P09 (Portage, MI; different colony room) (Fig. 4*B*; main effect of barrier; F_(1,132.21)_ = 10.69, p = 1.38×10^−3^). Rats from barrier P03 performed more magazine entries during the CS period (data not shown; F_(1,133.06)_ = 5.28, p = 0.023), but they did not differ from rats from barrier P09 on latency to or probability of magazine entries (data not shown; latency: F_(1,135.88)_ = 1.51, p = 0.22; probability: F_(1,133.51)_ = 0.49, p = 0.49). Rats from barrier P09, however, performed more lever presses with a shorter latency and higher probability than rats from barrier P03 (data not shown; number: F_(1,142.86)_ = 10.91, p = 1.12×10^−3^; latency: F_(1,136.15)_ = 10.01, p = 1.92×10^−3^; probability: F_(1,136.87)_ = 17.35, p = 5.48×10^−5^). Inclusion of other variables as random effects, such as time of year and identity of the experimenter, did not influence the significance of vendor or barrier fixed effects for any of the tests reported here, and none of the included random effects reached or approached statistical significance (data not shown).

**Figure 5:**
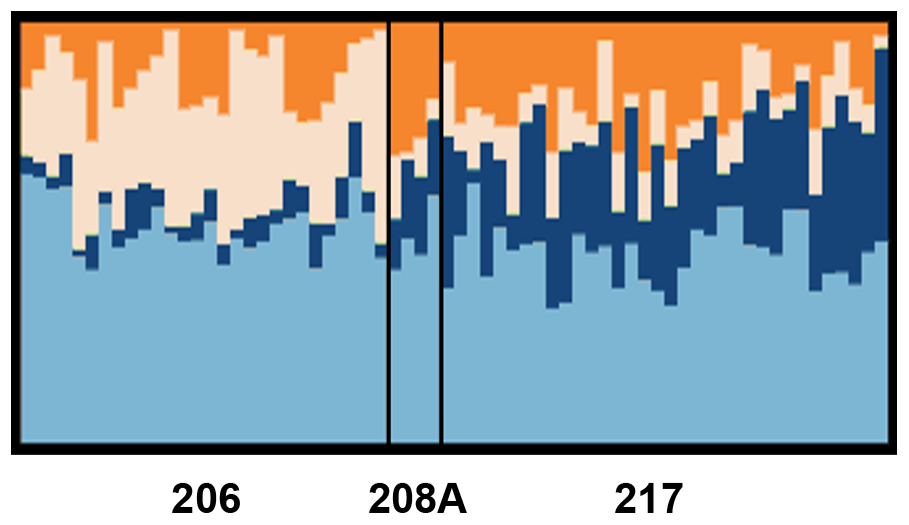
Population structure among Harlan colonies. Each Sprague-Dawley (SD) rat from different colonies of Harlan (206, Haslett, MI; 207A, Frederick, MD; and, 217, Indianapolis, IN) is represented by a vertical bar, which is partitioned into four colors, representing the four contributing populations that were identified by *structure*. The colony rooms are labeled below the panel.

Next, we investigated whether there was evidence of population stratification among the SD rats from different Harlan colonies by using the program *structure* [25]. In this model, a value of K>1 indicates significant population structure, and in our sample the optimal parameter for K was 4, suggesting that the different colonies are genetically distinct populations (Fig. 5). The rats from the colonies at Frederick, MD (208A), and Indianapolis, IN (217), looked somewhat similar while the rats from Haslett, MI (206) appeared to be outliers. Interestingly, this is consistent with the observed phenotypic differences (Fig. 4).

## Discussion

It has been demonstrated previously that sign-tracking behavior differs between two inbred strains of rats (i.e., Lewis and Fischer) [27]; however, this study is the first to demonstrate that the incidence of sign- and goal-tracking behavior also reliably varies within an outbred stock of rats at the level of vendors and individual colonies. Specifically, SD rats acquired from Harlan displayed more sign-tracking behavior, whereas rats acquired from Charles River displayed more goal-tracking behavior. In addition, rats acquired from individual colonies of each vendor differed significantly in the form of PCA behavior: Charles River rats acquired from barrier P03 displayed more goal-tracking behavior than rats acquired from barrier P09, and Harlan rats acquired from barriers 206, 217, and 208A all showed significantly different levels of sign-tracking behavior. This is also the first study that we are aware of that explicitly demonstrates population structure among outbred rats, an observation that supports the hypothesis that genetic differences may account for the observed behavioral differences.

The methods we employed do not allow us to determine to what degree genetic or environmental variables affected the incidence of sign- and goal-tracking among rats acquired from different vendors and colonies. However, there is evidence that genetic factors can influence PCA behavior. For example, rat lines selectively bred on the basis of locomotor response to novelty differ reliably in PCA behavior [28]. Environmental factors can also have enduring effects on PCA behavior. For example, rats that experience early life stress in the form of maternal separation tend to exhibit more sign-tracking behavior when tested as adults [29]. Conversely, rats that are reared in an enriched environment show reduced sign-tracking behavior compared to those raised in isolation [30].

As previously mentioned, the divergent phenotypes between both vendors and colonies may also be the result of genetic factors. By using genotyping-by-sequencing and analyzing population structure, we demonstrated that rats acquired from Harlan colonies 208A (Frederick, MD) and 217 (Indianapolis, IN) are more genetically similar to each other than rats acquired from 206 (Haslett, MI), which is consistent with our observed phenotypic differences. That is, rats acquired from 208A and 217 are more phenotypically similar to each other than to rats acquired from 206. Both population bottlenecks and random genetic drift can lead to these observed genetic differences among population isolates. Such genetic differences are inevitable among finite breeding populations; for example, genetic drift has been previously reported in certain outbred stocks of OF-1 [31] and CLFP [32] mice. In addition, genetic and phenotypic differences have been observed among outbred CD-1 mice acquired from different breeding facilities [33]. Although genotyped microsatellite markers are used by vendors to determine genetic variation within barriers (e.g., the International Genetic Standardization program at Charles River), the screens are only performed on a small number of rats, and typically there are several years between tests [34].

Though a detailed analysis of environmental differences between the populations tested in this study was not possible, there are several potential sources of environmental variation that could have affected PCA behavior. For instance, animal transportation is stressful to rats, and air transportation increases corticosterone (CORT) [35]. Previously, it has been demonstrated that CORT release is positively correlated with sign-tracking behavior in rats and mice [36], [37]. There are several reasons why we believe that transportation stress did not underlie the observed alterations in PCA behavior here. First, the aforementioned transportation-induced increase in CORT normalized within one week, which coincided with our period of acclimation and handling. Second, all of our rats were shipped via truck, which is considered a less stressful method of transportation. Third, colonies P03 and P09 (Charles River) are both located in the nearby city of Portage, MI, yet the rats, which travelled the same distance and under similar conditions, still differed in PCA behavior. Both basal and evoked stress responses vary between SD rats supplied by either Harlan or Charles River [8], [38], however, and we cannot discount the fact that SD rats acquired from different vendors vary in their responses to stress experienced in the colonies or during transport, which may have affected subsequent PCA behavior. Every effort is made to ensure that different colonies within the same company are essentially identical, so it is unlikely that environmental enrichment contributed in a significant way to the PCA differences observed between colonies. The effects of early-life experiences, however, cannot be completely excluded. For example, different staff members handle the animals in different colonies, which could result in varying stress levels between animals. Studies of environmental factors affecting rodent behavior consistently demonstrate that experimenter identity is one of the strongest sources of variance in standardized behavioral measures [39], [40].

Reliable variation in complex behaviors has not been fully investigated across paradigms. Between January 2002 and July 2005, it was estimated that 85% of all publications in the PubMed database involving rats used outbred stocks [41], which may show reliable variation between vendors and colonies. When maximal phenotypic variation in complex behaviors in outbred rat stocks is desired, it may be advantageous to acquire rats from different sources. However, care must be taken with such an approach not to mistakenly attribute a biological correlation between two traits simply because random genetic drift or other extraneous causes happened to enrich them both within a particular colony. Though restriction of experimental subjects to a single colony avoids the potential confound of such spurious correlations, it also risks reducing phenotypic variation. In either case, knowledge of how behavior varies among outbred stocks acquired from both vendors and colonies can assist in the design and success of experiments.

## Supporting Information

Figure S1 **Pretraining session influence on Pavlovian conditioned approach index scores.** PCA scores (mean+S.E.M.) averaged across the last two training sessions for rats who received one (n = 255) or two (n = 135) pretraining sessions.
(TIF)

## References

[1] Silver LM (1995) *Mouse Genetics: Concepts and Applications*. New York: Oxford University Press.

[2] Festing MF (2010) Inbred strains should replace outbred stocks in toxicology, safety testing, and drug development. Toxicol Pathol 38: 681–690. 20562325 10.1177/0192623310373776

[3] Glick SD, Shapiro RM, Drew KL, Hinds PA, Carlson JN (1986) Differences in spontaneous and amphetamine-induced rotational behavior, and in sensitization to amphetamine, among Sprague-Dawley derived rats from different sources. Physiol Behav 38: 67–70. 3786503 10.1016/0031-9384(86)90133-2

[4] Pollock DM, Rekito A (1998) Hypertensive response to chronic NO synthase inhibition is different in Sprague-Dawley rats from two suppliers. Am J Physiol 275: R1719–1723. 9791095 10.1152/ajpregu.1998.275.5.R1719

[5] Buhimschi IA, Shi SQ, Saade GR, Garfield RE (2001) Marked variation in responses to long-term nitric oxide inhibition during pregnancy in outbred rats from two different colonies. Am J Obstet Gynecol 184: 686–693. 11262473 10.1067/mob.2001.110448

[6] Bueno A, de Olmos S, Manzini F, Desmond NL, de Olmos J (2003) Strain and colony differences in the neurotoxic sequelae of MK-801 visualized with the amino-cupric-silver method. Exp Toxicol Pathol 55: 287–294. 14703775 10.1078/0940-2993-00327

[7] Miller FP, Cox RH Jr, Maickel RP (1968) Intrastrain difference in serotonin and norepinephrine in discrete areas of rat brain. Science 162: 463–464. 5683055 10.1126/science.162.3852.463

[8] Pecoraro N, Ginsberg AB, Warne JP, Gomez F, la Fleur SE, et al (2006) Diverse basal and stress-related phenotypes of Sprague Dawley rats from three vendors. Physiol Behav 89: 598–610. 16935312 10.1016/j.physbeh.2006.07.019

[9] Oliff HS, Coyle P, Weber E (1997) Rat strain and vendor differences in collateral anastomoses. J Cereb Blood Flow Metab 17: 571–576. 9183296 10.1097/00004647-199705000-00012

[10] Fuller DD, Baker TL, Behan M, Mitchell GS (2001) Expression of hypoglossal long-term facilitation differs between substrains of Sprague-Dawley rat. Physiol Genomics 4: 175–181. 11160996 10.1152/physiolgenomics.2001.4.3.175

[11] Pare WP, Kluczynski J (1997) Differences in the stress response of Wistar-Kyoto (WKY) rats from different vendors. Physiol Behav 62: 643–648. 9272677 10.1016/s0031-9384(97)00191-1

[12] Bert B, Fink H, Sohr R, Rex A (2001) Different effects of diazepam in Fischer rats and two stocks of Wistar rats in tests of anxiety. Pharmacol Biochem Behav 70: 411–420. 11701214 10.1016/s0091-3057(01)00629-3

[13] Marosi M, Rakos G, Robotka H, Nemeth H, Sas K, et al (2006) Hippocampal (CA1) activities in Wistar rats from different vendors. Fundamental differences in acute ischemia. J Neurosci Methods 156: 231–235. 16621009 10.1016/j.jneumeth.2006.03.010

[14] Honndorf S, Lindemann C, Tollner K, Gernert M (2011) Female Wistar rats obtained from different breeders vary in anxiety-like behavior and epileptogenesis. Epilepsy Res 94: 26–38. 21277170 10.1016/j.eplepsyres.2010.12.012

[15] Swerdlow NR, Platten A, Kim YK, Gaudet I, Shoemaker J, et al (2001) Sensitivity to the dopaminergic regulation of prepulse inhibition in rats: evidence for genetic, but not environmental determinants. Pharmacol Biochem Behav 70: 219–226. 11701191 10.1016/s0091-3057(01)00598-6

[16] Robinson TE, Flagel SB (2009) Dissociating the predictive and incentive motivational properties of reward-related cues through the study of individual differences. Biol Psychiatry 65: 869–873. 18930184 10.1016/j.biopsych.2008.09.006 PMC2737368

[17] Flagel SB, Clark JJ, Robinson TE, Mayo L, Czuj A, et al (2011) A selective role for dopamine in stimulus-reward learning. Nature 469: 53–57. 21150898 10.1038/nature09588 PMC3058375

[18] Saunders BT, Robinson TE (2011) Individual variation in the motivational properties of cocaine. Neuropsychopharmacology 36: 1668–1676. 21471956 10.1038/npp.2011.48 PMC3138662

[19] Yager LM, Robinson TE (2010) Cue-induced reinstatement of food seeking in rats that differ in their propensity to attribute incentive salience to food cues. Behav Brain Res 214: 30–34. 20416342 10.1016/j.bbr.2010.04.021 PMC2910199

[20] Saunders BT, Robinson TE (2010) A cocaine cue acts as an incentive stimulus in some but not others: implications for addiction. Biol Psychiatry 67: 730–736. 20045508 10.1016/j.biopsych.2009.11.015 PMC2849872

[21] Peterson BK, Weber JN, Kay EH, Fisher HS, Hoekstra HE (2012) Double digest RADseq: an inexpensive method for de novo SNP discovery and genotyping in model and non-model species. PLoS One 7: e37135. 22675423 10.1371/journal.pone.0037135 PMC3365034

[22] Elshire RJ, Glaubitz JC, Sun Q, Poland JA, Kawamoto K, et al (2011) A robust, simple genotyping-by-sequencing (GBS) approach for high diversity species. PLoS One 6: e19379. 21573248 10.1371/journal.pone.0019379 PMC3087801

[23] Li H, Durbin R (2009) Fast and accurate short read alignment with Burrows-Wheeler transform. Bioinformatics 25: 1754–1760. 19451168 10.1093/bioinformatics/btp324 PMC2705234

[24] Li H, Handsaker B, Wysoker A, Fennell T, Ruan J, et al (2009) The Sequence Alignment/Map format and SAMtools. Bioinformatics 25: 2078–2079. 19505943 10.1093/bioinformatics/btp352 PMC2723002

[25] Pritchard JK, Stephens M, Donnelly P (2000) Inference of population structure using multilocus genotype data. Genetics 155: 945–959. 10835412 10.1093/genetics/155.2.945 PMC1461096

[26] Meyer PJ, Lovic V, Saunders BT, Yager LM, Flagel SB, et al (2012) Quantifying individual variation in the propensity to attribute incentive salience to reward cues. PLoS One 7: e38987. 22761718 10.1371/journal.pone.0038987 PMC3382216

[27] Kearns DN, Gomez-Serrano MA, Weiss SJ, Riley AL (2006) A comparison of Lewis and Fischer rat strains on autoshaping (sign-tracking), discrimination reversal learning and negative auto-maintenance. Behav Brain Res 169: 193–200. 16469395 10.1016/j.bbr.2006.01.005

[28] Flagel SB, Robinson TE, Clark JJ, Clinton SM, Watson SJ, et al (2010) An animal model of genetic vulnerability to behavioral disinhibition and responsiveness to reward-related cues: implications for addiction. Neuropsychopharmacology 35: 388–400. 19794408 10.1038/npp.2009.142 PMC2794950

[29] Lovic V, Keen D, Fletcher PJ, Fleming AS (2011) Early-life maternal separation and social isolation produce an increase in impulsive action but not impulsive choice. Behav Neurosci 125: 481–491. 21688886 10.1037/a0024367

[30] Beckmann JS, Bardo MT (2012) Environmental enrichment reduces attribution of incentive salience to a food-associated stimulus. Behav Brain Res 226: 331–334. 21945300 10.1016/j.bbr.2011.09.021 PMC3687775

[31] Hoger H (1992) Genetic drift in an outbred stock of mice. Jikken Dobutsu 41: 215–220. 1577082

[32] Papaioannou VE, Festing MF (1980) Genetic drift in a stock of laboratory mice. Lab Anim 14: 11–13. 6928500 10.1258/002367780780943015

[33] Aldinger KA, Sokoloff G, Rosenberg DM, Palmer AA, Millen KJ (2009) Genetic variation and population substructure in outbred CD-1 mice: implications for genome-wide association studies. PLoS One 4: e4729. 19266100 10.1371/journal.pone.0004729 PMC2649211

[34] Charles River Laboratories International, Inc. (2011) International Genetic Standardization (IGS) Program. Retrieved from http://www.criver.com/SiteCollectionDocuments/rm_rm_r_IGS.pdf. Accessed 16 March 2013.

[35] Shim S, Lee S, Kim C, Kim B, Jee S, et al (2009) Effects of air transportation cause physiological and biochemical changes indicative of stress leading to regulation of chaperone expression levels and corticosterone concentration. Exp Anim 58: 11–17. 19151507 10.1538/expanim.58.11

[36] Tomie A, Lincks M, Nadarajah SD, Pohorecky LA, Yu L (2012) Pairings of lever and food induce Pavlovian conditioned approach of sign-tracking and goal-tracking in C57BL/6 mice. Behav Brain Res 226: 571–578. 22026925 10.1016/j.bbr.2011.10.021 PMC3412063

[37] Flagel SB, Akil H, Robinson TE (2009) Individual differences in the attribution of incentive salience to reward-related cues: Implications for addiction. Neuropharmacology 56 Suppl 1139–148. 18619474 10.1016/j.neuropharm.2008.06.027 PMC2635343

[38] Turnbull AV, Rivier CL (1999) Sprague-Dawley rats obtained from different vendors exhibit distinct adrenocorticotropin responses to inflammatory stimuli. Neuroendocrinology 70: 186–195. 10516481 10.1159/000054475

[39] Chesler EJ, Wilson SG, Lariviere WR, Rodriguez-Zas SL, Mogil JS (2002) Influences of laboratory environment on behavior. Nat Neurosci 5: 1101–1102. 12403996 10.1038/nn1102-1101

[40] Crabbe JC, Wahlsten D, Dudek BC (1999) Genetics of mouse behavior: interactions with laboratory environment. Science 284: 1670–1672. 10356397 10.1126/science.284.5420.1670

[41] Chia R, Achilli F, Festing MF, Fisher EM (2005) The origins and uses of mouse outbred stocks. Nat Genet 37: 1181–1186. 16254564 10.1038/ng1665

