# Supplementary figures and images for: Variation in the Form of Pavlovian Conditioned Approach Behavior among Outbred Male Sprague-Dawley Rats from Different Vendors and Colonies: Sign-Tracking vs. Goal-Tracking
Source: PLoS One. 2013 Oct 1;8(10):e75042. doi: 10.1371/journal.pone.0075042 (PMC3787975; doi:10.1371/journal.pone.0075042)

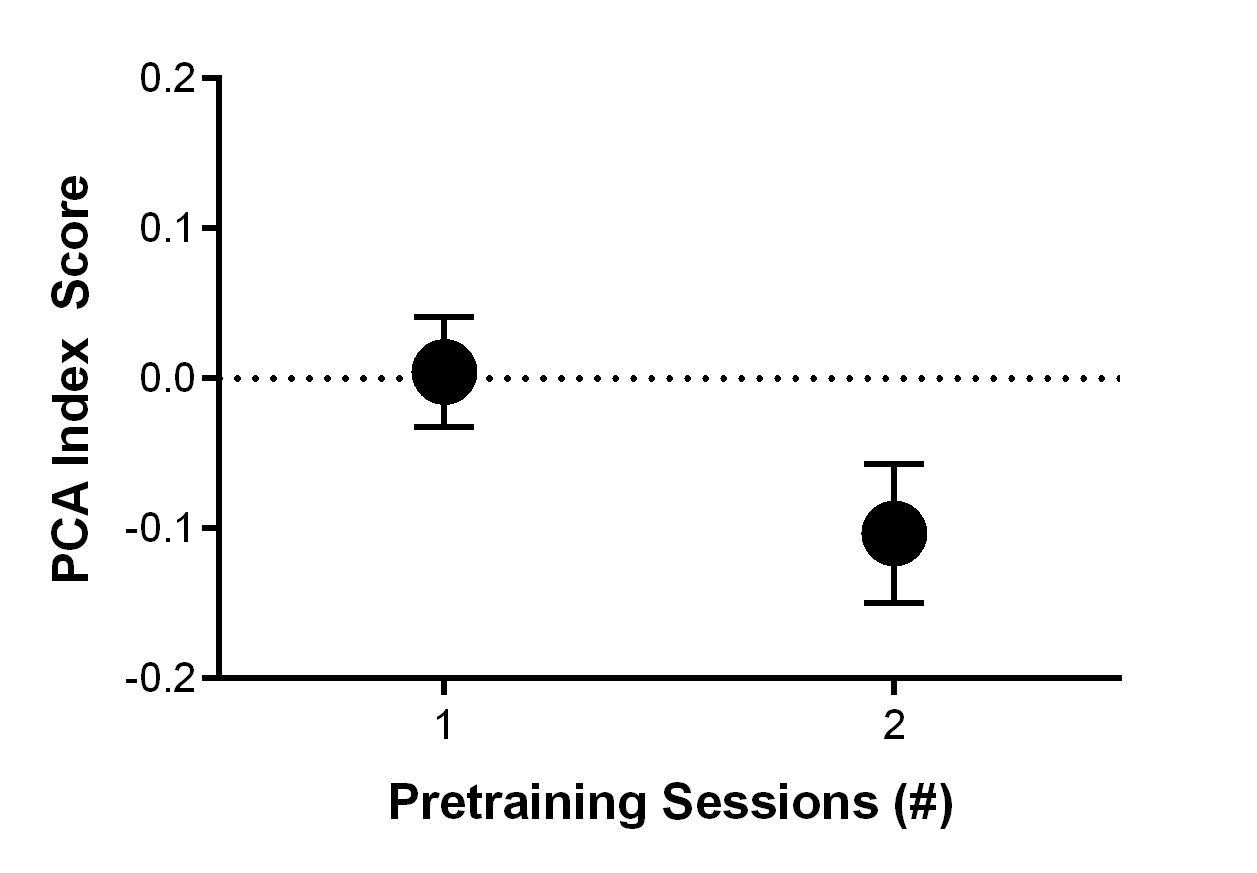

Supplement: Figure S1 — Pretraining session influence on Pavlovian conditioned approach index scores. PCA scores (mean+S.E.M.) averaged across the last two training sessions for rats who received one (n = 255) or two (n = 135) pretraining sessions. (TIF) [file pone.0075042.s001.tif]
